# IMPROVED PLANAR KIDNEY ACTIVITY CONCENTRATION ESTIMATE BY THE POSTERIOR VIEW METHOD IN ^177^LU-DOTATATE TREATMENTS

**DOI:** 10.1093/rpd/ncw046

**Published:** 2016-06-07

**Authors:** Tobias Magnander, Johanna Svensson, Magnus Båth, Peter Gjertsson, Peter Bernhardt

**Affiliations:** 1 Department of Radiation Physics, Institute of Clinical Sciences, Sahlgrenska Academy at University of Gothenburg, SE-413 45 Gothenburg, Sweden; 2 Department of Medical Physics and Biomedical Engineering, Sahlgrenska University Hospital, SE-413 45 Gothenburg, Sweden; 3 Department of Oncology, Institute of Clinical Sciences, Sahlgrenska Academy at University of Gothenburg, SE-413 45 Gothenburg, Sweden; 4 Department of Clinical Physiology, Sahlgrenska University Hospital, SE-413 45 Gothenburg, Sweden

## Abstract

The aims of this study were to determine how different background regions of interest (ROIs) around the kidney represent true background activity in over- and underlying tissues in ^177^Lu-DOTA-octreatate (^177^Lu-DOTATATE) treatments and to determine the influence of the background positions on the kidney activity concentration estimates by the conjugate view (ConjV) and posterior view (PostV) methods. The analysis was performed in single-photon emission computed tomography (SPECT) images of 20 patients, acquired 24 h post injection of a ^177^Lu-DOTATATE treatment, by a computer algorithm that created planar images from the SPECT data. The ratio between the activity concentration in the background and the true background varied from 0.36 to 2.08 [coefficient of variation (CV) = 25–181 %] and from 0.44 to 1.52 (CV = 16–70 %) for the right and left kidneys, respectively. The activity concentration estimate in the kidneys was most accurate with the PostV method using a background ROI surrounding the whole kidney, and this combination might be an alternative planar method for improved kidney dosimetry in the ^177^Lu-DOTATATE treatments.

## INTRODUCTION

Neuroendocrine tumours (NETs) are originating from several different organs, where the most common are the intestine, pancreas and lungs. Despite the originating diversity of NETs, they share many common features such as expression of somatostatin receptors (sstr)^(1)^. For NETs with high sstr expression, the radiolabelled somatostatin analogue ^177^Lu-DOTA-octreatate (^177^Lu-DOTATATE) has become an attractive therapeutic option^(2, 3)^. ^177^Lu is a beta-emitter with a maximum energy of 498 keV, half-life of 6.73 d, and emits a 208 keV gamma photon, which is used for gamma camera imaging^(4)^. One main critical organ for ^177^Lu-DOTATATE treatment is the kidney, with an absorbed dose limit of 23–27 Gy^(5, 6)^. Due to the limited kidney toxicity observed, it is thought that the absorbed dose limit can be raised^(7–10)^. However, increasing the absorbed dose limit requires accurate absorbed dose estimates. Today, the most common method for kidney dosimetry is to estimate the activity concentration in planar gamma camera images by the conjugate view method^(10–19)^. However, this method is connected with several uncertainties. One of the least investigated uncertainties is the placement of the region of interest (ROI) for the background used for subtracting the over- and underlying tissue activities from the organ ROI. In the clinically performed planar dosimetry studies, different locations are used for the background ROI, which might be one reason for different mean absorbed doses presented^(6, 8, 10, 12–19)^.

The aims of this study were to determine how well background ROIs for the conjugate view technique represent true background activity in over- and underlying tissues (TB) and to compare the accuracy in the activity concentration estimates in the kidneys between the conjugate view (ConjV) and posterior view (PostV) methods.

## MATERIALS AND METHODS

### Patients and administration of ^177^Lu-DOTATATE

This retrospective study was approved by the Regional Ethical Review Board in Gothenburg and performed in accordance with the Declaration of Helsinki and national regulations. The need for written informed consent was waived. From March 2006 to December 2011, 51 patients with advanced NETs were treated with ^177^Lu-DOTATATE at Sahlgrenska University Hospital in Gothenburg, and in the present study, the single-photon emission computed tomography (SPECT) data from the first 20 consecutive patients were analysed.

In the treatment with ^177^Lu-DOTATATE, the patients received an average amount of 7.5 GBq, given as a 30-min intravenous infusion, co-administered with kidney-protective amino acids (2.5 % lysine and 2.5 % arginine in 1 L of 0.9 % NaCl; rate of infusion 250 ml h^−1^).

### Gamma camera measurements

In this study, the quantitative analyses were performed in SPECT images, and the planar images were only used for visual inspection and validation of the method used to convert SPECT data into planar images (described below). The gamma camera used for generating planar and SPECT/CT images at 24 h post injection (h.p.i.) was a Millennium VG Hawkeye (General Electric Medical Systems, Milwaukee, WI, USA), equipped with medium-energy parallel-hole collimator, and a computed tomography (CT) system.

**Figure 1.**
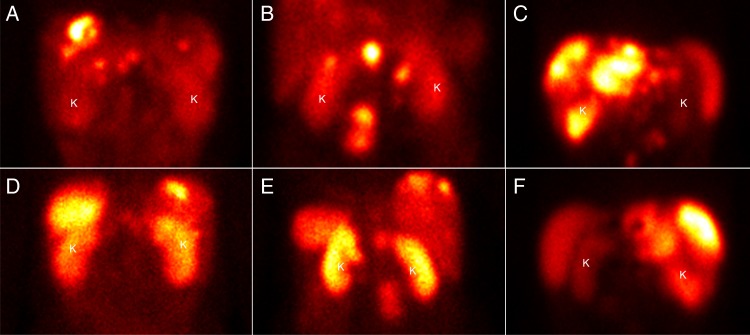
The anterior (**A–C**) and posterior (**D–F**) planar gamma camera images of three patients treated with ^177^Lu-DOTATATE: (A, D), (B, E) and (C, F). The kidney (K) is most easily visualised in the posterior view.

**Figure 2.**
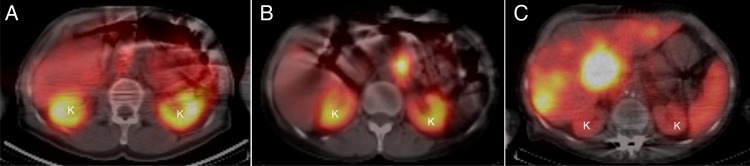
Transversal SPECT/CT midsections of the kidneys (K) in three patients treated with ^177^Lu-DOTATATE. **A**–**C** correspond to the same patients investigated with planar image as in Figure 1.

Planar anteroposterior (AP) and posteroanterior (PA) whole body scans were performed with scanning time 10 cm min^−1^ and a 20 % energy window over the 208 keV photon peak. The matrix size was 256 × 1024 with a pixel size of 2.21 mm. Examples of typical planar gamma camera images are shown in Figure 1. The SPECT image acquisition used the same energy setting as above and was performed with a 30 s frame time duration for 120 projections. The matrix size was 128 × 128 with a pixel size of 2.21 mm and a slice thickness of 4.42 mm. The CT images were acquired using a tube voltage of 140 kV, 2.5 mAs, and a rotation speed of 2.6 rpm. The matrix size was 256 × 256 with a pixel size of 2.21 mm and a slice thickness of 4.42 mm. The CT matrix size was converted into a matrix size of 128 × 128 and fused with the SPECT images. SPECT reconstruction was performed by the ordered subset expectation maximum (OSEM) algorithm with 2 iterations, 10 subsets, and post-filtering. Some examples of transversal SPECT/CT images are shown in Figure 2.

### Automatic activity concentration estimate of true background and estimated background

The theoretical requirement for using the ConjV and PostV methods for activity estimates in 2D images is that all activity should be located in the organ of interest, i.e. the kidney in this study. However, in reality, there will always be activity in over- and underlying tissues. In the ConjV and PostV methods, a background ROI somewhere beside the organ ROI is used to estimate the over- and underlying tissue activities. To evaluate the accuracy of differently located background ROIs for estimation of the mean activity concentration in the over- and underlying tissues, the activity concentrations in the volumes of interest (VOIs) for the background ROIs and the true background, i.e. the mean activity concentration in the VOI for the over- and underlying tissues, were measured in SPECT images. The VOIs for the background ROIs and the true background were generated by PhONSAi—the Physics, Oncology & Nuclear medicine research image platform at the Sahlgrenska Academy.

**Figure 3.**
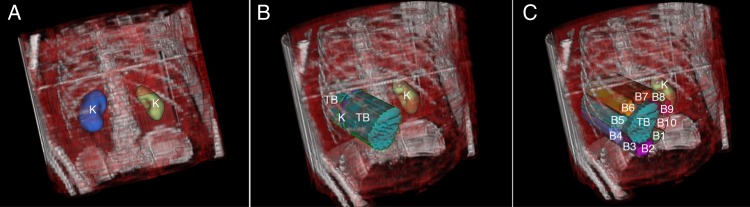
The automatic segmentation method of the (**A**) kidney VOIs (K), (**B**) the true background activity VOIs (TB), and the background VOIs for the kidney (1–10).

Subtracting a kidney VOI from a column VOI generated the true background VOI (TB). A segmentation algorithm delineated the kidney VOI (Figure 3a). To create the column VOI, the kidney VOI was projected onto the 2D plane and backprojected to 3D (Figure 3b). The length restriction of the column VOI was generated by level set thresholding of the SPECT image where a threshold of 5 counts was used to separate the patient body from the surrounding air. Thereby the column length was equal to the distance from the anterior to posterior body surfaces (i.e. equal to the patient thickness) (Figure 3b).

To determine the mean activity concentration that contributed to measured counts in the background ROIs, a similar method as described above was applied. Totally, 10 parallel background VOIs were created around the TB, with a distance of 2 voxels from the TB. The width of the background VOIs was 4 voxels, and the length was determined from the angle around the kidney, i.e. first the centre position of the kidney VOI projection was determined, and then the angle to each pixel in the backgrounds VOIs was determined. The pixels that were located within an angle between 0 and 36° were assigned to B1, 36–72° to B2 and so on to B10 with 324–360° (Figure 3c). An additional background ROI surrounding the whole kidney, B11, was also created. All the background ROIs were backprojected into 3D background column VOIs. The activity concentration was compared between the background VOIs and the TB by determining the background-to-true background ratio (BTR). The mean and the coefficient of variation (CV), i.e. the standard deviation of the BTR divided by the corresponding mean BTR, were calculated.

### Determination of the kidney activity concentration by the conjugate view and posterior view methods

To compare the accuracy in activity concentration estimates in the kidneys between the ConjV and PostV methods, attenuated planar kidney and background ROIs were created using the SPECT images and an attenuation map. The attenuation map for ^177^Lu was created from the CT:

(1) $$\mu (x,y,z)=\mu_{w}+\text{HU(}x,y,z)\frac{\mu_{w}}{1000},$$

where *µ*_w_ is the effective attenuation coefficient for 208 keV photons in water, and HU is the Hounsfield value. To validate that counts in the created planar kidney and background ROIs resemble the counts in planar scans, the counts in the SPECT-created ROIs were compared with similarly positioned ROIs in the planar scans. The kidney-to-background ratios (KBRs) for the two methods were compared for all patients. This was done by determining the mean and the CV of the ratio between the KBRs for the two methods.

For each background ROI, the net counts in the anterior view (*A*_net_) and posterior view (*P*_net_) in the kidney ROIs were calculated by subtracting the counts in the background ROI from the counts in the kidney ROI, where the background ROI was scaled for resembling the over- and underlying tissue volumes. The relative number of kidney ROIs with negative net counts for the ConjV method (CNC), i.e. negative *A*_net_ and/or negative *P*_net_, and the PostV method (PNC), i.e. negative *P*_net_, was calculated for the right and left kidneys.

For the ConjV method, the activity concentration in the kidney was calculated by the following equation:

(2) $$\text{A}{\text{C}}_{\mathrm{ConjV}}=\frac{\mu \times t_{k}\times \sqrt{A_{\mathrm{net}}\times P_{\mathrm{net}}}}{e^{-\frac{\mu \times t_{p}}{2}}\times \left(e^{\frac{\mu \times t_{k}}{2}}-e^{-\frac{\mu \times t_{k}}{2}}\right)\times v_{k}},$$

where *t*_k_ is the thickness of the kidney in the anterior to posterior direction, and *t*_p_ is the patient thickness in the anterior to posterior direction. These thicknesses were measured in the transversal CT slice in the midsection of the kidney. *µ* is the effective attenuation coefficient for ^177^Lu in water, and *v*_k_ is the volume of the kidney.

For the PostV method, calculation of the activity concentration in the kidney was performed by the following equation:

(3) $$\text{A}{\text{C}}_{\mathrm{PostV}}=\frac{P_{\mathrm{net}}}{v_{k}}\times e^{\mu \times d_{k}}\times \frac{t_{k}\times \mu }{1-e^{-\mu \times t_{k}}},$$

where *d*_k_ is the distance to the kidney from the patient's back, as measured in the transversal CT slice in the midsection of the kidney.

The relative kidney activity concentrations between the ConjV method and the SPECT quantification and between the PostV method and the SPECT quantification were calculated for the left and right kidneys in 20 consecutive patients treated with ^177^Lu-DOTATATE. Paired two-sided *t*-test was used for testing of statistically significant differences (*p* < 0.05) between the ConjV and PostV methods.

## RESULTS

The created algorithm implemented into the PhONSAi platform for analysing the influence of the position of the background ROI on planar activity estimate was stable and performed well in all patients. The SPECT-generated ROIs were validated to resemble the corresponding planar ROIs; the mean of the ratio between the KBRs of the two methods was 1.03, which was not statistically different from unity. The comparison generated a CV of 13 %, which was demonstrated (by repeated repositions of the ROIs) to be due to the challenge of positioning the ROIs equally in the two image systems and hence not affecting the evaluation performed in the SPECT images.

The comparison of the activity concentration in the background VOIs and the TB VOI revealed low accuracy and precision for most positions of the background ROIs (Table 1). For these ROIs, the BTRs varied from 0.36 to 2.08 for the right kidney and from 0.44 to 1.52 for the left kidney. The corresponding CVs ranged from 16 to 181 % and from 16 to 70 %, respectively. Of the 10 standard background ROIs, i.e. B1-B10, number B6 had a high accuracy, 1.01; however, the CV was high (47 %), and the number of negative net counts was 15 % for the ConjV method. No other standard ROI resembled the true background activity concentration better than this; in most cases, there was severe over- or underestimation of the activity concentration. The overestimation of the true background activity concentration logically increased the risk of obtaining negative net counts in the kidney ROI. The highest number of negative net counts was 45 % (B8) and 50 % (B9) for the right and left kidneys, respectively. In contrast, the background ROI that surrounded the whole kidney had high accuracy with a slightly lower CV. The mean BTRs were 1.0 and 0.97 for the right and left kidneys, respectively. The corresponding CVs were 39 and 24 %, respectively.

**Table 1.** The ROI background to true background ratio (BTR), the CV of the BTR and the relative number of kidney ROIs with negative net counts for the ConjV method (CNC) and the PostV method (PNC).

ROI	Right kidney				Left kidney			
	BTR	CV	CNC	PNC	BTR	CV	CNC	PNC
		(%)	(%)	(%)		(%)	(%)	(%)
B1	0.73	51	10	5	0,44	23	0	0
B2	0.53	38	10	0	0.44	16	0	0
B3	0.36	16	0	0	0.50	15	0	0
B4	0.44	25	0	0	0.70	37	10	0
B5	0.62	28	0	0	0.91	60	15	5
B6	1.01	47	15	5	1.16	63	35	0
B7	1.59	87	35	35	1.22	66	35	5
B8	2.08	181	45	35	1.52	70	40	40
B9	1.24	53	30	10	1.49	65	50	50
B10	0.81	44	15	0	0.91	44	15	5
B11	1.00	39	5	0	0.97	24	5	0

**Figure 4.**
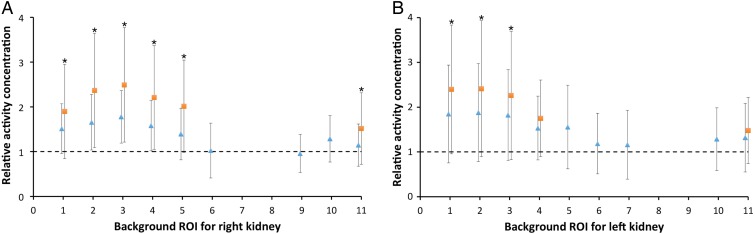
The relative kidney activity concentration between the ConjV method and the SPECT quantification (orange squares), and the PostV method and the SPECT quantification (blue triangles). **A** and **B** show the results for the right and left kidneys, respectively. The mean values and standard deviations are shown, and the stars indicate statistically significant differences between the ConjV and PostV methods. When data for the relative activity concentration are missing, the number of kidneys with negative net counts is more than 10 %.

The kidney activity concentration estimate by the ConjV and PostV methods was performed for background ROIs for which the relative number of negative net counts was 10 % or less. For the ConjV method, six and five background ROIs for the right and left kidneys, respectively, satisfied this restriction. This number was increased for the PostV method, having nine background ROIs for both the kidneys satisfying this restriction. In this analysis of the kidney activity concentration estimate, the PostV method resembled the true activity best (Figure 4). The pairwise *t*-test between the methods showed statistically significant differences in 9 of 11 evaluations. The accuracy for the ConjV method was poor in most cases, with relative activity concentration ratios ranging from 1.52 to 2.49 and from 1.48 to 2.41 for the right and left kidneys, respectively. The corresponding CVs ranged from 39 to 70 % and from 49 to 63 %, respectively. The highest accuracy and the lowest variability were obtained with the surrounding background ROI.

With the PostV method, a good accuracy was obtained for several background ROIs. For the right kidney, five backgrounds with the PostV method obtained higher accuracy than the best accuracy with the ConjV method. The corresponding number was four for the left kidney. The surrounding background ROI had high accuracy in both the kidneys, with relative activity concentration ratios of 1.15 and 1.32 for the right and left kidneys, respectively. The corresponding CVs were 41 and 58 %, respectively.

## DISCUSSION

The results in this study demonstrate the problematic situation for accurate kidney activity concentration estimate in planar images, and that the placement of the background ROI is highly involved in the poor accuracy and precision in the activity concentration estimate. In this study, the clinical situation where the operator positions a small ROI close to the kidney, with the aim that this position should resemble the activity concentration in the over- and underlying tissues for the organ of interest, was evaluated. However, the result demonstrated that differently positioned small background ROIs poorly resembled the true background. In many situations, the background position underestimated the true background up to 2-fold. These locations were caudal to the kidneys, which is a common location for background ROIs^(10–19)^. With an underestimated background subtraction, the activity concentration will be overestimated, and the calculated absorbed dose will be too high, reaching the absorbed dose limit too early. This might result in an interruption of the radionuclide therapy and thereby a non-optimised treatment with ^177^Lu-DOTATATE. Nonetheless, despite a low accuracy, the caudal position had the highest precision, which might make it possible to improve the accuracy by applying a calibration factor. Such an approach would be of interest for further studies with more patients involved. In this study, the background analysis was based on a total of 20 patients, which was sufficient for discriminating between the ConjV and PostV methods, but for high accuracy in a population-based calibration factor, a larger patient material would be necessary.

In contrast to the caudal position of the backgrounds ROIs, the cranial backgrounds ROIs overestimated the activity concentration in the over- and underlying tissues. Furthermore, the precision of the cranial position was extremely poor, with CV up to 180 %. The cranial backgrounds ROIs will not only underestimate the kidney activity concentration, but they may also result in negative net counts, making the calculations of the activity concentration impossible for some patients. The reasons for the high overestimate of the background concentration are the high organ uptake in the liver and spleen and the high uptake in tumours, often located in the liver and in the immediately cranial surrounding of the kidneys. In the gamma camera scans, these high-uptake areas are easily observed (Figure 1). In the authors' earlier studies, the kidney ROI has therefore been restricted to the inferior part of the kidney, and a caudal position of the background ROI has been used^(10, 15, 19)^. Such an approach will remove the most variable activity concentration part of both the kidney ROI and the background ROI, and it could be expected that the accuracy will be improved. Nevertheless, future studies have to determine the accuracy and precision for such a design.

In this study, a new approach for background determination was used by applying a background ROI that surrounds the whole kidney, i.e. a background ROI design similar to what is used in dynamical renal studies with ^99m^Tc-diethylene triamine pentaacetic acid^(20)^. This novel approach had high accuracy in estimating the true activity concentration. Furthermore, the precision was rather good, which makes the design an alternative for background ROIs also in absorbed dose estimate in radionuclide therapy.

Initially, it was the ambition in this work to manually outline the kidney ROIs in the planar images. However, as described above, the large variability in the kidney overlap from organs and tumours hampered the visibility of the kidney border, making it most challenging to correctly outline the kidney ROI (Figure 2). To reduce such high operator dependency on the performed analysis, it was decided to create the ROIs by automatic segmentation of the kidney and project it into the 2D plane. In this way, the operator dependence was removed. Nevertheless, operator dependence is a significant contributor to the accuracy and precision in the activity concentration estimate and would be of beneficial value to study further^(21, 22)^.

The conjugate view method is the most used planar method for activity estimates^(10–19)^. However, in the 20 planar gamma camera scans collected in this study, it was demonstrated that the visibility of the kidneys was superior in the posterior scans (in Figure 1, three scans are shown). Furthermore, the SPECT/CT images indicated that the overlapping activity was located more on the ventral side of the kidney and very seldom dorsal to the kidney (Figure 2). This indicated that the PostV method might be an alternative method for activity estimates, and therefore the method was included in this study. In line with the expectations, the result demonstrated that the PostV method had higher accuracy and precision than the ConjV method. While the PostV method had several situations where the accuracy was almost perfect, the ConjV method always had a pronounced overestimation of the activity concentration. This overestimation of the ConjV method is in line with the results of He *et al.*^(22)^, who investigated the accuracy and precision of the ConjV method in a phantom study. They used a caudal background ROI position that yielded an overestimation of 116 % with a poor precision of 63 %, i.e. similar result as in the present study for small ROIs. In the present study, it is interesting to note that the approach of using a larger surrounding background ROI improved both the accuracy and precision and might be the background ROI of choice, at least when the whole kidney is outlined. However, as previously mentioned, the whole kidney is most challenging to correctly outline, and He *et al*. presented an improved accuracy (57 %) and precision (44 %) when the kidney ROI did not include overlapping organs^(22)^. Nevertheless, the results of the present study demonstrate that the PostV method has a high accuracy also for a whole outlined kidney. One advantage of the PostV method is that only the posterior view is used, in which the kidney most often is easily visualised and therefore can be outlined in a straightforward way^(23)^. Furthermore, the highest activity concentration is located ventrally to the kidney and thereby has a smaller influence on the required background subtraction.

The method used for background correction in this study was to subtract the counts in a normalised background ROI, i.e. the measured counts in the background ROI were scaled to resemble the same volume as the over- and underlying tissues. The use of the normalised background ROI is an improvement from not using rescaling. A further improvement might be to calculate the mean activity concentration in the background ROI by the ConjV method and subtract it from the corresponding mean activity concentration in the kidney ROI^(14, 24)^. Such an approach is simple and straightforward when the activity concentration is uniformly distributed, but this is not the case for the activity concentration distributions in patients under ^177^Lu-DOTATATE treatments. In contrast, the activity concentration distribution is highly non-uniform and would require higher-ordered attenuation correction that might not be solvable for pure planar dosimetry. Nevertheless, since the result of this study showed a general overestimation of the activity, further analysis and improvement of the background subtraction method would be valuable.

As mentioned above, the poor accuracy of the planar methods might be possible to correct by, e.g., the use of a population-based calibration factor. In contrast, the poor precision of the planar methods is more puzzling to solve. In this study, some of the uncertainties might be attributed to the quantitative SPECT method used, having its own intrinsic variation. However, the precision in quantitative SPECT is rather good in comparison with the planar methods; e.g. He *et al.* have reported that the CV of quantitative SPECT can be less than 5 %^(22)^. Another source of uncertainty in the analysis is that the evaluation was performed in a SPECT image that was projected into planar images for analysis. This source of uncertainty was investigated by comparing the SPECT-generated ROIs with similar ROIs in planar images collected immediately after the SPECT collection. The mean ratio between these planar/SPECT ROIs was 1.03 with a CV of 13 %; the ratio was not statistically different from unity. The rather high variation in the ratio is most probably due to methodological uncertainties in the validation process, i.e. difficulties to obtain the same ROI positions in the two image systems. Lastly, since the operator dependency was removed with the used methodology, the uncertainties in this study might have been underestimated, and not overestimated. Further studies have to be performed to quantify these effects.

## CONCLUSION

This study demonstrates that when the whole kidney ROI is outlined, the PostV method is superior to the ConjV method for planar quantification of activity concentrations; the use of a surrounding background ROI is a robust method for obtaining good accuracy; and the combination might be an alternative planar method for improved kidney dosimetry in ^177^Lu-DOTATATE treatments.

## FUNDING

This work was supported by the Swedish Cancer Society (12 0686), the Swedish Radiation Safety Authority (SSM2015-2371), the King Gustav V Jubilee Clinic Cancer Research Foundation (2014:33) and the Swedish Federal Government under ALF agreement (ALFGBG-440231).
